# Deficiency of the Promyelocytic Leukemia Protein Fosters Hepatitis C-Associated Hepatocarcinogenesis in Mice

**DOI:** 10.1371/journal.pone.0044474

**Published:** 2012-09-11

**Authors:** Kerstin Herzer, Anna Carbow, Svenja Sydor, Jan-Peter Sowa, Stefan Biesterfeld, Thomas-Georg Hofmann, Peter-Robert Galle, Guido Gerken, Ali Canbay

**Affiliations:** 1 Department of Gastroenterology and Hepatology, University Hospital, Essen, Germany; 2 Spital STS Ag, Department of Surgery, Zweisimmern, Swizerland; 3 Department of Cytopathology, University Hospital of Düsseldorf, Düsseldorf, Germany; 4 Research Group Cellular Senescence, Deutsches Krebsforschungszentrum, Deutsches Krebsforschungszentrum- Zentrum für Molekulare Biologie der Universität Heidelberg Alliance, Heidelberg, Germany; 5 1^st^ Department of Medicine, University Medicine of the Johannes Gutenberg University, Mainz, Germany; 6 Gereral-, Viszeral and Transplantation Surgery, University Hospital, Essen, Germany; Kanazawa University, Japan

## Abstract

Overwhelming lines of epidemiological evidence have indicated that persistent infection with hepatitis C virus (HCV) is a major risk for the development of hepatocellular carcinoma (HCC). We have recently shown that HCV core protein mediates functional inactivation of the promyelocytic leukemia (PML) tumor suppressor pathway. However, the role of PML in HCC development yet remains unclear. To clarify the function of PML in liver carcinogenesis and HCV-associated pathogenesis we crossed PML-deficient mice with HCV transgene (HCV-Tg) expressing mice and treated the resulting animals with DEN/Phenobarbital, an established protocol for liver carcinogenesis. Seven months after treatment, livers were examined macroscopically and histologically. Genetic depletion of the tumor suppressor PML coincided with an increase in hepatocyte proliferation, resulting in development of multiple dysplastic nodules in 100% of the PML-deficient livers and of HCCs in 53%, establishing a tumor suppressive function of PML in the liver. In animals expressing the HCV-transgene in PML-deficient background, HCC development occurred even in 73%, while only 7% of their wildtype littermates developed HCC. The neoplastic nature of the tumors was confirmed by histology and expression of the HCC marker glutamine synthetase. Several pro- and antiapoptotic factors were tested for differential expression and liver carcinogenesis was associated with impaired expression of the proapoptotic molecule TRAIL in PML-deficient mice. In conclusion, this study provides first *in vivo* evidence that the tumor suppressor PML acts as an important barrier in liver carcinogenesis and HCV-dependent liver pathology.

## Introduction

Liver cancer is the fifth most common cancer worldwide and the third most common cause of cancer mortality. Hepatocellular carcinoma (HCC), which accounts for 80%–90% of primary liver tumors, is characterized by a very poor prognosis and is associated with high mortality [1]. Chronic Hepatitis C and associated liver cirrhosis represent major risk factors for HCC development, being implicated in more than 70% of HCC cases worldwide with increasing incidence in the western world [2]. About 170 million people are infected with the hepatotropic Hepatitis C virus (HCV). HCV is a small RNA virus coding for a limited number of four structural and six nonstructural polypeptides, which regulate HCV replication and encapsulation of the viral genome [3]. Several viral proteins have been implicated in liver carcinogenesis with emphasis on the HCV core protein. For example, HCV core protein has been described to facilitate cellular transformation [4]. Among cellular host factors, which interact with HCV core is the tumor suppressor protein p53, a key regulator of the cellular response to genotoxic stress and antiviral response [5]. Despite our growing knowledge about HCV-host cell interaction, the molecular mechanisms which contribute to HCV-mediated transformation and carcinogenesis are still incompletely understood. Several studies using transgenic mouse models indicate that HCV is directly involved in hepatocarcinogenesis, although other factors such as continuous inflammation or environmental factors seem also to play a role [6]]. The downstream events of the HCV protein expression in the transgenic mouse HCC model are segregated into two pathways. One is augmented oxidative stress in the absence of inflammation along with the attenuation of some scavenging systems in the putative preneoplastic stage with steatosis in the liver. The other pathway is the alteration in cellular gene expression and intracellular signalling, including the mitogen-activated protein kinase cascade [7].

By focusing on the cellular function of HCV core protein we recently uncovered a previously unidentified link between HCV core and promyelocytic leukemia-nuclear bodies (PML-NBs). We found that HCV core protein targets PML-NBs and inactivates the PML tumor suppressor pathway through interfering with the apoptosis-inducing function of PML isoform IV [8]. PML-NBs are present in almost every human cell type analyzed so far and appear as discrete nuclear domains in immunofluorescence. PML exerts potent growth suppressive and apoptosis-inducing activities [9], and PML-deficient mice and cells exhibit defects in multiple apoptosis pathways [10]. Furthermore, PML deficiency has been linked to increased susceptibility to viral pathogens [11], [12]. A large number of proteins with diverse functions have been found to localize to PML-NBs and their central role in multiple cellular processes such as proliferation, apoptosis, and regulation of transcription is well established [13]. Moreover, comprehensive studies have shown that the PML protein is frequently lost in human cancers of various origins [14]. So far, a functional role for PML in HCC has not been defined.

In this study, we used transgenic mice with liver specific expression of HCV RNA corresponding to the full-length open reading frame (ORF) of the hepatitis C virus [15]. These mice were crossbread with PML−/− mice [16] to achieve a HCV-transgenic and PML−/− genotype. For both parental strains, no spontaneous liver tumor development has been described so far.

Our data presented herein show that PML-deficiency gives way to increased sensitivity of liver cells to carcinogen and HCV-associated HCC development. This indicates that the tumor suppressor PML is an indispensable factor in the complex interplay of liver tumor development in HCV-dependent liver carcinogenesis.

## Materials and Methods

### Animals and Genotyping

PML−/− mice (within a 129Sv genetic background) (kindly provided by Hans Will, Heinrich Pette-Institut, Hamburg, Germany) were generated by Pier Paolo Pandolfi (Beth Israel Deaconess Medical Center, Boston, USA) and have been described previously [16]. HCV transgenic FL-N/35 mice (within a C3H/C57BL6 genetic background) (kindly provided by Ula Hibner, IGMM, Montpellier, France) were generated by Herve Lerat (INSERM, Paris, France) and Stanley M. Lemon (UTMB, Galvestone, USA) [15]. The two mouse strains were crossed and the following genotypes were used for this study: (1)WT; PML+/+, (2)HCV; PML +/+, (3)WT; PML−/− and (4)HCV; PML−/−.

Genotyping was performed with the following primers: PML: R: 5′ TTG GAC TTG CGC GTA CTG TC-3′, F1∶ 5′-TTT CAG TTT CTG CGC TGC C-3′, F2∶ 5′- CGA CCA CCA AGC GAA ACA -3. HCV: NS4b forward MWG Biotech AG # 27-5072 1/4 5′-TAT TGC CTG ACA AGA GGC AGT GTG GTT ATC-3′, NS4b reverse MWG Biotech AG # 27-5072 2/4 5′-GAT GAA ATT CCA CAT GTG CTT TGC CCA G-3′.

PCR was performed in a standard thermocycler and analyzed on 2% agarose gels.

### Aminotransferase Levels

About 100 µL of blood was collected from the tail vein. Alanine aminotransferase (ALT) and aspartate aminotransferase (AST) were measured in the Institute of Clinical and Laboratory Medicine at the University Hospital Mainz by standard procedures.

### Chemical Hepatocarcinogenesis

Transgenic mice as well as WT mice were treated according to a hepatocarcinogenesis protocol consisting of tumor-initiation with diethylnitrosamine (DEN) and tumor-promotion with phenobarbital (PB) as described [17]. In brief, DEN (Sigma) was dissolved in PBS and given twice i.p. at a dose of 5 µg/g bodyweight at the age of 7 and 10 days post partum. Initiation was followed by PB treatment (Sigma) in a concentration of 0.05% continuously added to drinking water. Only male mice were included into the study. Fifteen mice of each group were sacrificed at the indicated time point after the beginning of the treatment with PB and were further analysed. Age matched WT and transgenic littermates which were left untreated were used as controls. Liver and body weight were determined. At month 7, all remaining mice were killed, since the incidence of hepatocellular carcinoma increased and a high percentage of transgenic mice started to suffer from cachexia or tumor burden.

### Histopathology and Histology

All mice in the carcinogenesis protocol were subjected to a complete necropsy and tissues were immediately fixed in 4% neutral-buffered formaldehyde, embedded in paraffin, sectioned at 2 µm and stained with hematoxylin/eosin (HE) by standard methods. For assessment of liver pathology at least eight representative sections of each liver were prepared and evaluated by a senior pathologist in a blinded fashion. Liver pathology was scored into preneoplastic lesions (dysplastic nodules) and hepatocellular carcinomas.

Gomori, methenamine silver, and periodic acid-Schiff were performed to allow histological evaluation of liver fibrosis (staging), inflammatory activity and tumor (grading), respectively. The histology of specimens was independently and blindely assessed by two board certified pathologists.

In addition, we sacrificed the corresponding untreated transgenic mice at the age of 2 or 7 months. A complete autopsy with inspection of the liver, lung, kidneys, intestine, spleen and heart for macroscopically visible tumors was performed. Suspicious tissue lesions were examined histopathologically.

### Histology and Immunohistochemistry

In addition to the above mentioned staining techniques, slides were immunostained for Ki67 (monoclonal rabbit, 1∶100 dilution; NeoMarkers) using an automated staining system with an iView DAB kit (Ventana, Tucson, AZ). All sections were counterstained with hematoxylin. The whole section was evaluated for the number of positive hepatocytes, and pictures were taken from representative high-power fields.

Immunohistochemical stainings of all liver specimens were performed in order to investigate expression of glutamin synthetase and proliferating cell nuclear antigen (PCNA) using the Envision staining method (Dako, Denmark) as described earlier [18]. The antigen retrieval was carried out by steamer treatment in pH 6 citrate buffer and mouse-anti-glutamine synthetase antibodies (diluted 1∶500) and rabbit-anti PCNA antibody (diluted 1∶1000) was applied, respectively. Specimens were then incubated with anti-mouse or anti-rabbit envision secondary antibodies conjugated with horse raddish peroxidase (HRP) and immunostainings were developed using diaminobenzidine as a substrate.

### TUNEL Staining

Cell death was detected by staining cell nuclei with DNA strand breaks (TUNEL technology) using the *in situ* Cell Death Detection Kit, Fluorescein (Roche), according to the manufacturer’s instructions. Counterstaining and further procedure was performed as described above.

**Figure 1:**
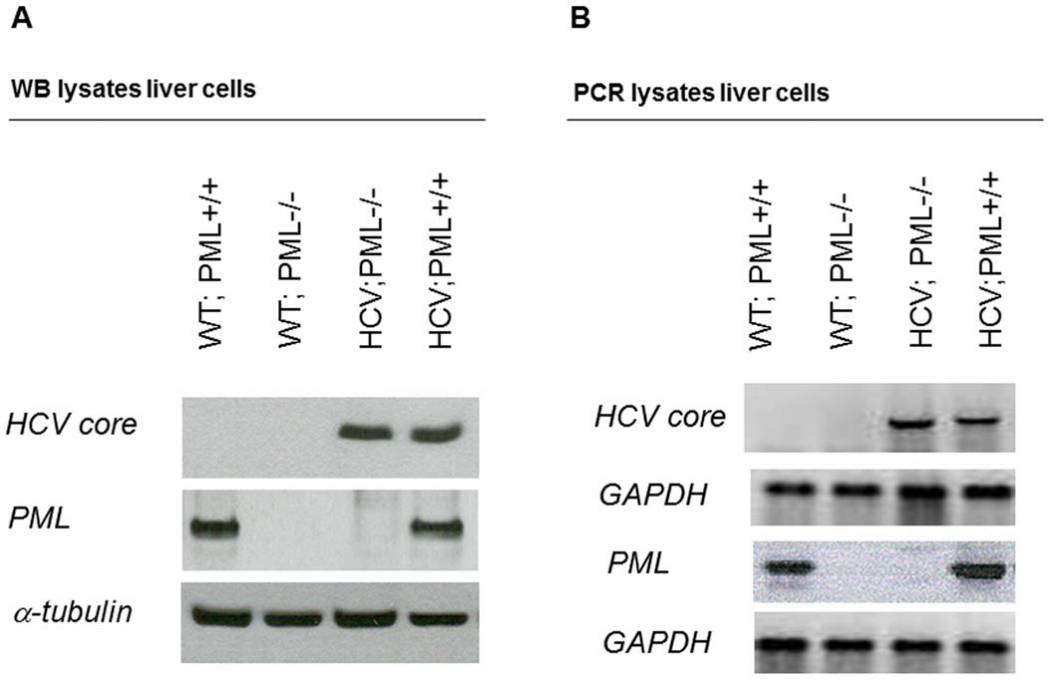
Characterisation of PML-deficient HCV-transgenic mice. (A) Whole liver extracts derived from 4 weeks old wild-type and HCV-PML+ mice were analysed by immunoblot analysis for the protein expression of PML and HCV-core as well as tubulin (loading control). (B) Whole liver extracts from 4 weeks old PML-HCV+ mice as well as WT mice were analysed for the mRNA expression levels of PML and HCV core by quantitative RT-PCR.

**Figure 2:**
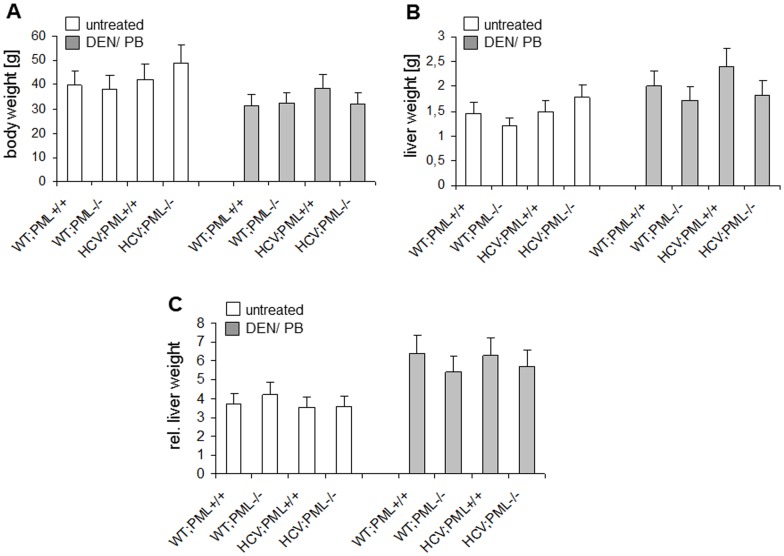
PML-expression influences liver weight. (A) Body weight, (B) liver weight as well as (C) liver/body weight ration of all mouse strains, either treated or not, was determined.

### Tissue Lysis and Western Blotting

About 20 mg of shock-frozen liver tissue were minced, transferred into ice-cold lysis buffer containing 20 mM Tris-HCl (pH 8.0), 5 mM EDTA, 0.5% Triton-X 100, and 1× Protease inhibitor cocktail (Roche Diagnostics, Mannheim, Germany) and incubated on ice for 15 min. Western blotting was performed as described [19]. Immunodetection was performed using the following primary antibodies: PML (Santa Cruz Biotechnology; H-238 and PG-M3), HCV core (MA1-080 obtained from ABR, Golden, CO, USA), TRAIL (Novus Biologicals, Littleton, USA) and anti-tubulin (Sigma). Peroxidase-conjugated species-specific secondary antibodies (Santa Cruz Biotechnology) were used at a dilution of 1∶10000. Western blots were performed for at least two mice at each age indicated.

### RNA Isolation

For isolation of total RNA, 20 mg of shock frozen liver tissue were homogenized in 1 mL TRI-Reagent (Sigma), and further isolated according to the manufacturers instructions. RNA concentration was measured in a NanoDrop photometer (Peqlab, Erlangen, Germany) and 1 µg of total RNA was subjected to reverse transcription using oligo-dT primers. Isolated DNA and complementary DNA (cDNA) were used for PCR (see above) and real time (RT) PCR approaches, respectively.

**Figure 3:**
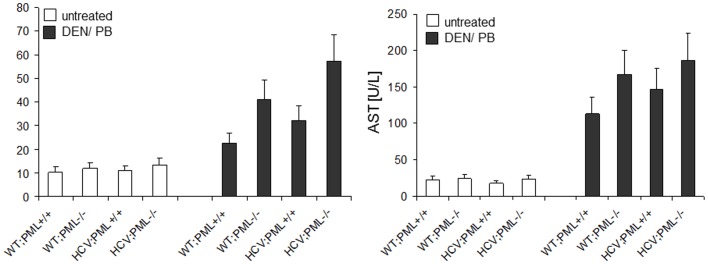
PML-expression influences transaminases. Serum AST and ALT levels of treated (white bars) and untreated (grey bars) mice. Mean levels of 15 mice per group ± SD are shown.

### RT Quantitative PCR

Relative target messenger RNA (mRNA) expression was analyzed by RT-quantitative PCR using the QuantiTect SYBR Green PCR Kit and QuantiTect primers (Quiagen, Hilden, Germany) for murine TRAIL, TRAIL-R, CD95, CD95R, NOXA, PUMA, Mcl-1, bcl-2, bcl-xL, survivin, XIAP, and Succinate dehydrogenase complex, subunit A (SDHA) as housekeeping gene. The relative increase in reporter fluorescent dye emission was monitored. The level of target mRNA, relative to SDHA, was calculated using the standard formula.

## Results

### Validation of PML-deficiency in the Liver of HCV-Tg Mice

After breeding *Pml* −/− mice to HCV-Tg mice, homozygous offspring were screened for deletion of PML and expression of the HCV-Tg. We analysed PML and HCV expression in liver lysates of 4-week-old mice (Fig. 1a). Immunoblotting demonstrated absent PML expression in liver lysates of PML −/− as well as PML −/−/HCV-Tg mice. In addition, RT-PCR showed loss of PML mRNA expression in total liver lysates of PML −/− mice in absence and presence of the HCV transgene (Fig. 1b). We used the following genotypes for further analysis: (1)WT; PML+/+, (2)HCV; PML +/+, (3)WT; PML−/− and (4)HCV; PML−/− (Fig. 1). Of each genotype, 15 male mice were submitted to the DEN/Phenobarbital hepatocarcinogenesis protocol [17] in order to achieve initiation of liver carcinogenesis during a considerable life span. The same number of mice of each group were left untreated as control. Mice were sacrificed at the age of 7 months.

**Figure 4:**
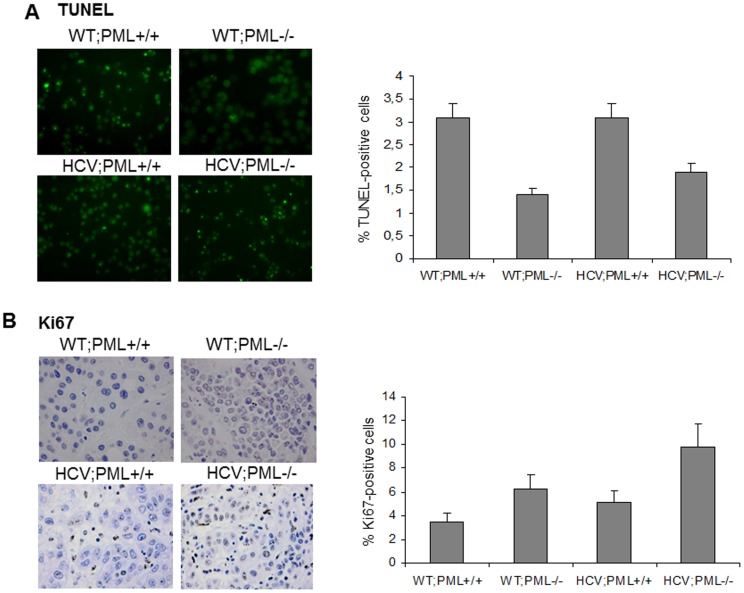
Apoptosis and proliferation in PML-deficient livers. (A) Liver sections were stained for DNA strand breaks by TUNEL assay. Five independent fields (40×) of five mice per genotype were quantified. The ratio of TUNEL-positive nuclei per 100 cells (TUNEL index) was calculated. (B) To assess hepatocyte proliferation Ki67 staining was performed. Five independent fields (40×) of five mice per genotype were quantifiend for Ki67-positive hepatocytes.

### Basal Liver Damage and Increased Apoptosis in PML-deficient HCV-transgenic Mice

The impact of PML deficiency in the presence or absence of the HCV-Tg for liver homeostasis after 7 months of treatment was assessed. In general, body weight of the mice was decreased in the DEN/Phenobarbital treated group, presumably due to tumor burden and cachexia (Fig. 2a). In contrast, liver weight was considerably higher in treated mice, which was more evident in PML-expressing animals and less in PML-deficient mice (Fig. 2b). HCV;PML+/+ and WT;PML+/+ animals showed under treatment an increased liver weight compared to age-matched PML-deficient and untreated controls, presumably due to deregulated apoptosis and regeneration, which may be compromised when the tumor suppressor PML is lacking (Fig. 2b). As a consequence, all animals showed under treatment an increase of liver/body weight ratio compared to age-matched untreated controls (Fig. 2c).

**Figure 5:**
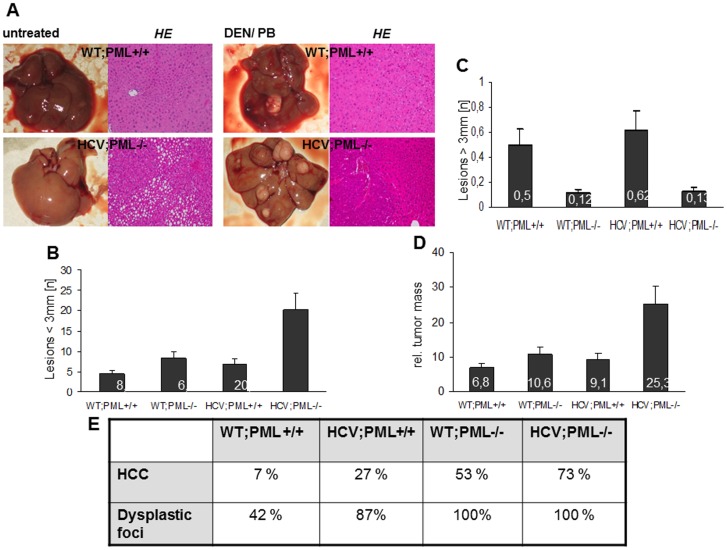
Liver tumors develop predominantly in HCV+PML- mice. Macroscopic inspection and HE staining of livers of all analysed mice revealed a spectrum of findings ranging from a macroscopically unremarkable (untreated) to a strongly nodular structure (PML-deficient with HCV-Tg) at the age of 7 months (A). Macroscopic analysis of all 4 DEN/PB-treated mouse strains as indicated, with 15 mice in every group. PML-deficient mice are more susceptible towards carcinogenic stimuli and develop dysplastic nodules evolving to carcinomas after 7 months. Tumorous lesions were macroscopically analysed and quantitatively evaluated as lesions <3 mm in (B) and >3 mm in (C). Relative tumor mass was calculated as number of lesions in relation to average size of lesions and liver mass (D). The data displayed in (B), (C) and (D) refer to the mice which displayed lesions. The number of mice displaying lesions *pro rata* 15 mice in every group is given as percentage of the livers showing at least one dysplatic focus or HCC in (E).

Aminotransferase levels were determined as a surrogate marker for liver tissue damage. As expected, liver enzymes were specifically increased in all animals treated with DEN/PB, whereas untreated mice showed comparably low transaminase concentrations (Fig. 3). Interestingly, PML-deficient mice exhibited a more than 4-fold increase in serum ALT levels and a 6–8-fold increase in Serum AST levels. In contrast, PML-deficient mice bearing the HCV-Tg displayed a non-significant but considerable higher concertration in both transaminases (Fig. 3). An increase in transaminases indicates a damage of liver cells, therefore these results suggest an additional liver damage in HCV;PML−/− mice.

**Figure 6:**
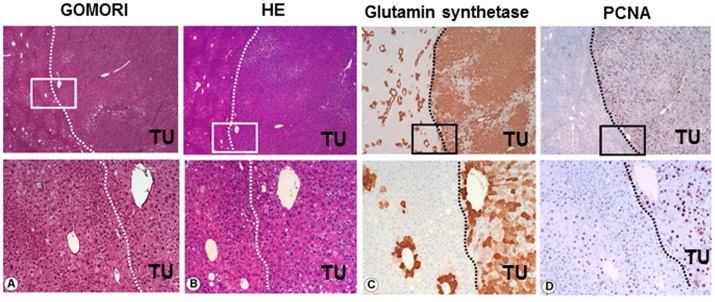
Development of liver cell tumors (HCC) in PML- HCV+ mice. The Gomori staining (A) and H&E (B) of the specimens demonstrate that no fibrotic changes have occurred during tumor development. Immunohistochemistry indicates a high expression of glutamine synthetase in most HCCs, however only pericentral expression was observed in the peritumorous tissue area (C). PCNA staining revealed a high proliferation rate in the HCC (D). The dashed border lines show the intersection between the HCC and the peritumorous tissue areas. The lower pictures were high magnification of the squared areas in the upper pictures in the figure legends.

We next asked whether this coincides with hepatocyte cell death. Therefore, we analyzed liver sections of 7-months-old untreated mice for DNA strand breaks using the TUNEL assay. Intact expression of PML was associated with more TUNEL-positive cells (Fig. 4a). Quantification demonstrated that the TUNEL-index was decreased in PML^−/−^ livers. Among PML-positive hepatocytes, independend of the HCV transgene status, TUNEL-index reached up to 3%. In contrast, liver sections of PML-deficient mice revealed hardly any or no TUNEL-positive hepatocytes (Fig. 4a).

Next we tested if reduced apoptosis activity in HCV;PML−/− mice was accompanied by increased hepatocyte proliferation by Ki67 staining. Ki67 expression was clearly increased in HCV;PML−/− hepatocytes (median: 9,8%) as well as in WT;PML−/− hepatocytes (median: 6,2%) when compared to WT-littermates. Furthermore, liver cells of mice expressing HCV-transgene also displayed a slight increase in proliferation when compared to PML-deficient wildtype mice (median: 5,1% versus 3,5%) (Fig. 4b). Taken together, our results indicate that both PML deficiency and HCV-transgene expression contribute to the strong proliferation rate of HCV;PML−/− hepatocytes.

Next, we analysed expression of inflammatory mediators in liver lysates, to determine if the increase in liver damage in PML−/− hepatocytes leads to an inflammatory response. No difference in IL-6 or TNFα mRNA expression in untreated mice was observed (data not shown). However, expression of both inflammation markers was increased after 7 months in DEN/PB treated mice, most likely due to the carcinogenesis treatment.

### PML Deficiency Potentiates Tumor Development in HCV Transgenic Mice

Deletion of the tumor suppressor PML did not induce spontaneous development of liver tumors up to an age of 7 months, independent of HCV transgene status. However, chemical induction of tumor development with DEN/PB treatment lead to a clear increase of liver tumors in HCV+;PML−/− animals when compared to identically treated WT mice (Fig. 5).

In all treatment groups, livers exhibited abnormal morphology. All livers displayed numerous pleomorphic and atypical hepatocytes and an altered, remarkably nodular liver structure, which was in contrast to livers of age-matched untreated WT mice (Fig. 5a). In addition to macroscopic quantification of tumors in mouse livers, we also measured the size of the individual lesions. To this end, five representative liver slides of every mouse of the four treatment groups as well as untreated control groups were subjected to morphometric analysis of the size of the tumor area. Interestingly, PML-negative livers displayed strikingly more lesions (median: 20±5) with smaller diameter (<3 mm) in average after treatment (Fig. 5b), while PML-positive mice developed relatively more lesions with a diameter >3 mm (Fig. 5c). Furthermore, average tumor mass was calculated in relation to liver mass and given as relative tumor mass in Fig. 5d. In WT mice the relative tumor mass counts for 6,8±1,1, whereas in HCV;PML−/− mice the relative tumor mass is 5-fold as high (25,3±6,1). Therefore, in PML-deficient mice not only the number of tumor lesions in total was considerably increased but also the cumulative tumor area was markedly larger.

To determine whether the observed tumors are indeed HCCs, livers were analysed by board certified pathologists. Histopathological evaluation of the livers after 7 months yielded at least one HCC in 73% of HCV;PML−/− mice. In contrast, only 7% of the WT;PML+/+ mice exhibited HCC formation (Fig. 5e).

WT;PML−/− mice developed at least one HCC in 53% of animals even in absence of the HCV-Tg and HCV;PML+/+ mice in 27%. Thus, PML-deficiency was associated with a significant increase in HCC incidence which was further aggravated by the HCV transgene.

However, after 7 months of treatment at least one dysplastic focus was found in livers of all PML-deficient mice. In contrast, only 42% of WT mice displayed at least one dysplastic focus.

The larger nodules found in PML-positive livers where either dysplasias or unspecific fibrotic nodules most likely due to regenerative processes. In contrast, PML-deficient livers develop a higher number of rather small dysplasias that develop to HCC at an early stage of growth. Untreated age-matched mice in the control groups revealed macroscopically normal livers.

In summary, we observed that PML-deficient mice were markedly more sensitive towards carcinogenic stimuli, which was further pronounced in presence of the HCV transgene.

Histologic analysis confirmed that all of the HCV;PML−/− mice displayed liver tumors at the age of 7 months (Fig. 4). Those tumors were characterized by cellular atypia, altered liver architecture with broadening of liver cell cords and loss of reticulin fibers (shown by Gomori staining; Fig. 6a). In addition, the proliferation rate was increased compared to non-tumorous areas, as a focal pattern of strong immunoreactivity for glutamine synthetase was observed (Fig. 6c) paralleled by more PCNA positive cells (Fig. 6d). Taken together the histological findings qualify the tumors as HCC.

### Liver Carcinogenesis is Accompanied by Downregulated TRAIL Expression

To obtain insight into the molecular mechanisms of tumor development in PML−/− livers with or without the HCV transgene, mRNA expression of several apoptosis-related factors was analysed.

**Figure 7:**
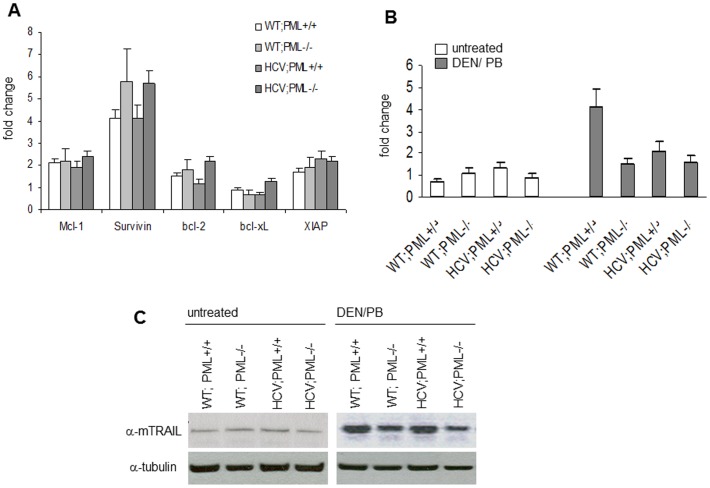
Expression of pro- and antiapoptotic genes. (A) mRNA expression analysis of several anti-apoptotic factors (Mcl-1, Survivin, Bcl-2, XIAP, Bcl-xL) by quantitative RT-PCR in the four treated transgenic groups. Boxes represent the average expression level of control. All values are normalized to SDHA mRNA expression. Standard deviation is indicated by error bars. (B) mRNA expression analysis of TRAIL by quantitative RT-PCR, comparing the four treated mouse strains versus untreated controls. Boxes represent the average expression level of control. All values are normalized to SDHA mRNA expression. Standard deviation is indicated by error bars. (C) Whole liver extracts derived from 7 months old mice were analysed by immunoblot analysis for the protein expression of TRAIL as well as tubulin (loading control). A representative of five independent experiments is shown.

Elevated transcript levels of survivin were detected in livers of 7 months old mice compared to untreated controls, with a non-significant but considerable increase in PML−/− livers only for survivin and bcl-2 (Fig. 7a). However, neither transcript levels of Mcl-1, Bcl-xL or XIAP were significantly different between treatment groups, whether expressing PML or the HCV transgene or not.

Differential expression of the proapoptotic genes NOXA, PUMA, CD95L/CD95R and TRAIL-R between treated mice versus untreated controls could not be found (data not shown).

However, the expression of the proapoptotic factor TRAIL, a protein of the TNF-family associated with hepatocyte apoptosis and carcinogenesis in the liver, exhibited enhanced induction upon treatment in both PML-expressing mouse strains. TRAIL was clearly induced in treated WT;PML+/+ mice which showed mild tumor growth after liver carcinogenesis. In contrast, TRAIL expression was low in the PML-deficient mice, independent of HCV (Fig. 7b). Furthermore, HCV-Tg expression also resulted in reduced TRAIL expression levels, indicating that HCV suppresses TRAIL expression *in vivo*. These findings were as well reflected on the protein level (Fig. 7c). Since TRAIL-expression was increased upon treatment in both PML-positive treatment groups, our findings suggest that TRAIL-regulation requires functional PML in this context. Furthermore, in accordance with our previous findings [8] this suggests that HCV core may inactivate PML partially and, thus, TRAIL-expression is compromised upon expression of the HCV-Tg.

## Discussion

HCC is recognized as one of the most common and most malignant cancers worldwide. The hepatitis C virus (HCV) counts as a major risk factor for the development of HCC [20], [21] and there is increasing experimental evidence to suggest that the virus plays a direct role in neoplastic transformation [22]. However, the molecular mechanisms causing this sequence of events are still poorly understood. In this study, we describe HCC development in HCV-Tg mice after depletion of the tumor suppressor PML. Our results suggest that: (I) PML protects against hepatocarcinogenesis, (II) PML-deficiency makes liver cells more susceptible towards carcinogenic stimuli, (III) HCV promotes carcinogenesis in the liver (IV) the oncogenic potential of HCV is supported by inactivation of PML.

The tumor suppressor PML is the essential structural organizer of nuclear multiprotein structures termed PML-NBs [23]. Studies in knockout mice and cells unraveled an essential pleiotropic role for PML in multiple p53-dependent and -independent apoptotic pathways [19], [24]. PML−/− mice and cells are protected from apoptosis triggered by a number of stimuli such as ionizing radiation, interferon, ceramide, Fas and TNF [10]. Recently we could show that the HCV core interferes with p53-dependent apoptosis in liver cells by interacting with PML and compromising its function [8]. Thus, we hypothesized that inactivity of PML could be a crucial madiator in HCV-associated carcinogenesis. Therefore, we generated HCV-transgenic PML-deficient mice by mating a mouse strain expressing the HCV transgene in the liver with PML−/− mice. In order to achieve tumor development in an appropriate time, the resulting mouse genotypes were treated with DEN/PB for half a year [17].

In our model, HCV-Tg livers exhibited a decrease in hepatocyte apoptosis rate, and an increase in proliferation and liver size. In HCV;PML−/− mice an enhanced proliferation rate of hepatocytes was observed. However, the decrease of apoptosis rate was not sufficient to compensate for the high grade of proliferation in the liver. Serum markers of liver cell damage, AST and ALT, were also significantly increased in PML-deficient mice with even higher values in presence of the HCV-Tg. Decreased body weight, increased liver size, high aminotransferase levels and high proliferation rate coincided with impressively pronounced hepatocarcinogenesis in HCV;PML−/− mice. Those mice displayed a considerably higher number of not only dysplastic nodules but also HCCs. However, both PML-deficient mouse strains developed more HCCs than the PML-expressing mouse strains. Interestingly, in PML-positive livers less but larger lesions were found, which were histologically characterized as mainly unspecific fibrotic nodules or early dysplasias. This might be explained by the fact that PML-expressing livers have a higher apoptosis rate and the process of damage, apoptosis and compensatory proliferation may lead to fibrotic changes due to regeneration. In case of PML-deficiency, apoptosis is severely compromised, proliferative processes prevail and carcinogenic processes take their track.

We detected liver tumors of varying size in all mouse strains after 7 months of treatment. Some liver tumors did not (yet) meet the histological criteria of HCC. However, morphology on a macroscopic and histological level of a number of tumors, combined with the expression of glutamine synthetase, confirmed them as HCC. The malignant character did not depend on the size of the lesions. In summary, HCC were heterogenous as corroborated by different patterns of morphology and immunohistochemistry. This argues against one particular molecular pathway involved in HCC formation in HCV-dependent carcinogenesis. In contrast it favours the notion that compensatory mechanisms underlie HCC formation in HCV-transgenic mice.

Under physiologic conditions, presence of viral proteins in the liver can cause an inflammatory response. Studies involving the use of transgenic mice have demonstrated that HCV and its proteins have the ability to induce fibrosis, either through direct interference with cell activation pathways or by indirect means, via the induction of steatosis [25]. The chronic inflammatory process of HCV infection could lead to increased mutagenesis in the regenerating hepatocytes, leading to a multi-step process of mutations finally presenting as HCC [26]. However, in auto-immune hepatitis, the occurrence of HCC is rare [27], raising doubts as to whether inflammation alone is able to lead to such a high incidence of HCC in patients infected with HCV. In line with that, in our model, we did not observe an increased invasion of inflammatory cells in treated or untreated transgenic mice. This is supported by unaltered IL-6 and TNF-alpha expression in untreated HCV-Tg mice. Mice treated with DEN/PB, though, have an increased expression of these inflammatory mediators which is somewhat expected in the context of the hepatocarcinogenesis protocol. But no differences between the different transgenic mouse groups were observed. Thus, contribution of inflammatory processes to the carcinogenic process may not be relevant in this model.

Previous experimental evidence suggests that the pathogenesis of HCC from HCV infection includes various viral protein–host cell interactions, which may play a direct role in the development of HCC [22], [28]. Perturbations in the cell cycle, combined with upregulation of oncogenes and loss of tumour-suppressor gene functions, may contribute to HCC development. Notably, HCV proteins have been shown to interact with these cellular pathways. We previously demonstrated that HCV core compromises apoptotic processes in liver cells by inhibition of PML [8]. Moreover, our herein presented data contribute the *in vivo* finding that HCV-transgenic mice are more susceptible towards carcinogenic stimuli. Thus, our results support a direct oncogenic implication of the hepatitis C virus which is in accordance with previous reports.

PML is a proapoptotic factor, and its deficiency favours an anti-apoptotic and pro-proliferative state. However, the absence of other anti-apoptotic factors may result in a hypoapoptotic environment, finally resulting in the outgrowth of a malignant cell population. The proapoptotic molecule TRAIL has gained attention for its ability to induce apoptosis in liver cancer cells without damaging normal liver cells [29], [30]. It may play an important role in preventing development and outgrowth of liver tumors. Moreover, TRAIL is partially regulated by PML in certain settings in the liver [19]. Our finding that TRAIL-levels are significantly reduced in PML-deficient animals suggests a possible contribution in the development of HCC in our model. While PML-positive livers do express TRAIL, the expression level is considerably compromised in presence of HCV. This is in line with our observation of HCV core compromising PML-function, leading to an impaired expression of PML-dependent proapoptotic factors. In addition to that, we were able to previously show a direct link between PML and TRAIL in HCC cell lines by RNAi silencing of PML, resulting in down-regulation of TRAIL expression in hepatoma cells. In addition, PML-deficient primary human hepatocytes fail to upregulate TRAIL upon IFN-alpha-treatment in contrast to their WT counterparts^19^. Furthermore, TRAIL and its receptors have been shown to be upregulated in the liver of HCV infected patients and PML is a molecule which, as well, is upregulated in inflammatory settings as viral hepatitis. The fact that TRAIL is also upregulated in an inflammatory context underlines PML-dependent regulation of TRAIL and supports a link between TRAIL and_PML. One may speculate that compromised expression of a death inducing molecule like TRAIL contributes to a privileged setting for outgrowth of liver tumor cells.

Taken together, this study provides the first *in vivo* evidence that deletion of the proapoptotic factor PML is accompanied by severely enhanced sensitivity of the liver towards carcinogenic stimuli and, as well, supports a direct oncogenic function of HCV driving HCC formation.
